# Case Series of Rare Uterine Smooth Muscle Tumors: Diagnostic Challenges and Clinical Implications

**DOI:** 10.7759/cureus.102302

**Published:** 2026-01-26

**Authors:** Mah Sheena Kalpaka Mohammed, Abdul Rahman El Kinge, Ehsan Ahmad

**Affiliations:** 1 Histopathology, NMC Royal Hospital, Sharjah, ARE; 2 Medicine, Sharjah University, Sharjah, ARE; 3 Hematology and Medical Oncology, NMC Royal Hospital, Sharjah, ARE

**Keywords:** leiomyosarcoma, smooth muscle tumors, stump, uterus, utrosct

## Abstract

Uterine smooth muscle tumors exhibit a wide range of pathological forms and biological behaviors. While leiomyomas are typically benign and frequently encountered, there exist rare variants and malignant counterparts that present notable diagnostic and therapeutic challenges. This case series explores a diverse group of uterine smooth muscle tumors, including leiomyomas with elevated mitotic figures, uterine tumors resembling ovarian sex cord tumors (UTROSCT), smooth muscle tumors of uncertain malignant potential (STUMP), myxoid leiomyosarcoma, and inflammatory leiomyosarcoma. Each case is described with a focus on histopathological features, immunohistochemical profile, clinical presentation, and corresponding management. The series underscores the importance of precise pathological categorization in guiding individualized treatment approaches.

## Introduction

Uterine smooth muscle neoplasms encompass a broad spectrum of tumors, ranging from common benign leiomyomas to aggressive malignancies. While conventional leiomyomas are well-recognized, rare subtypes and histologic variants - such as smooth muscle tumors of uncertain malignant potential (STUMP), myxoid leiomyosarcomas, and uterine tumors resembling ovarian sex cord tumors (UTROSCT) - can present significant diagnostic challenges due to overlapping morphological features [1]. Emerging studies have also highlighted molecular and immunohistochemical hallmarks that aid in distinguishing these entities, including alterations in p53, fumarate hydratase, and specific sex cord markers [2,3]. Accurate identification is crucial, not only for diagnosis but also for guiding prognostication and patient management, as clinical behavior ranges from indolent to highly aggressive [4]. This case series presents a spectrum of uncommon uterine smooth muscle tumors, emphasizing their histopathological, molecular, and clinical features to provide a practical framework for accurate diagnosis and optimal patient care.

## Case presentation

Case 1: Leiomyoma with elevated mitotic activity

**Figure 1:**
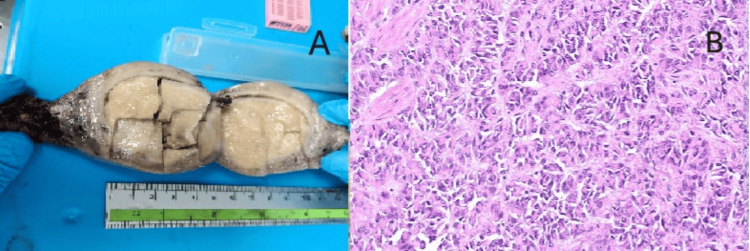
Gross, histopathological, and immunohistochemical features of the uterine tumor resembling ovarian sex cord tumor (UTROSCT). (A) Gross examination of the hysterectomy specimen showing a well-circumscribed, solid, tan-yellow intramural mass measuring approximately 6 cm in diameter within the uterine corpus. (B) Microscopic image revealing epithelioid tumor cells arranged in trabeculae, cords, and nests resembling ovarian sex cord-stromal tumors (hematoxylin and eosin, ×200).

A 45-year-old female presented with menorrhagia, pelvic fullness, and mild anemia for three months. Pelvic ultrasound revealed a hypoechoic intramural mass measuring 5.8 cm in diameter in the posterior uterine wall. MRI confirmed a well-circumscribed uterine mass consistent with a benign smooth muscle tumor, and a total hysterectomy was performed (Figure 1A). Histopathology demonstrated a well-circumscribed smooth muscle tumor with 7 mitoses per mm^2^, but without atypia or necrosis, consistent with leiomyoma with an elevated mitotic index. The patient has remained symptom-free for over a year.

Case 2: UTROSCT

A 38-year-old woman reported intermittent pelvic pain, bloating, and dysmenorrhea over the previous three months. MRI of the pelvis showed a 6 cm solid, well-defined intramural mass with intermediate T2 signal intensity and minimal enhancement, raising suspicion for a uterine neoplasm. Surgical excision was carried out.

Microscopy showed tumor cells arranged in cords and nests resembling ovarian sex cord tumors. Immunohistochemistry (IHC) revealed positivity for calretinin and inhibin, supporting the diagnosis of UTROSCT (Figure 1). The patient remains recurrence-free after 18 months of follow-up.

Case 3: STUMP

**Figure 2:**
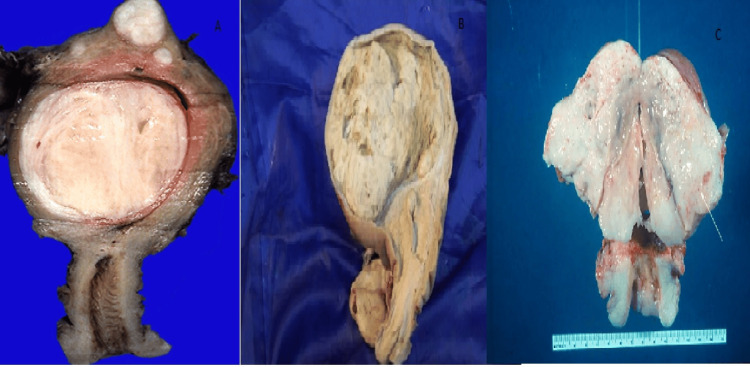
Gross photographs of hysterectomy specimens. Cut surface of the uterus displaying a well-circumscribed, firm, whorled, white-tan intramural/submucosal leiomyoma bulging into the endometrial cavity. (B) Cut surface of the uterus showing a partially circumscribed, tan-yellow mass with focal softening microscopically consistent with a smooth muscle tumor of uncertain malignant potential (STUMP). (C) Myxoid leiomyosarcoma appearing as a gelatinous, poorly circumscribed, lobulated mass with areas of hemorrhage and necrosis infiltrating the myometrium.

A 50-year-old postmenopausal woman underwent evaluation for mild postmenopausal bleeding. Transvaginal ultrasound incidentally identified a 4.5 cm well-defined heterogeneous mass in the posterior uterine wall. She underwent a total hysterectomy (Figure 2B).

Histology revealed unequivocal focal tumor cell necrosis, mild nuclear atypia, and a mitotic count of 8/10 HPF, insufficient for a leiomyosarcoma diagnosis. The tumor was classified as uterine spindle cell STUMP. The patient has remained stable with no recurrence at two-year follow-up.

Case 4: Myxoid leiomyosarcoma

A 60-year-old postmenopausal woman presented with the sudden onset of vaginal bleeding and rapid abdominal distension over two months. On physical exam, a large pelvic mass was palpable. MRI revealed a 10 cm, heterogeneously enhancing, infiltrative uterine mass with myxoid signal characteristics and areas of necrosis.

**Figure 3:**
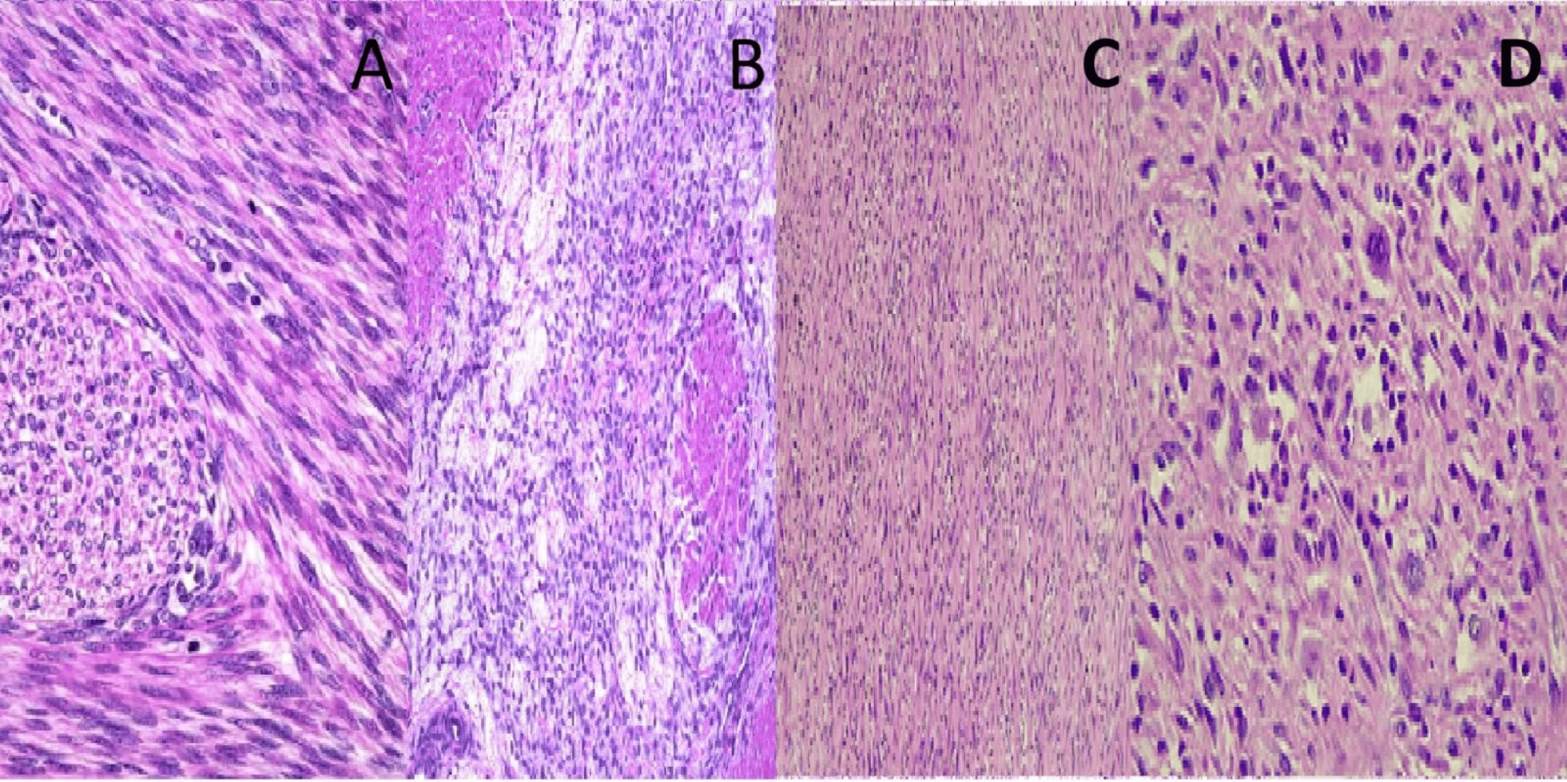
Photomicrography showing the histopathological spectrum of uterine smooth muscle tumors. (A) Photomicrograph showing a leiomyoma with increased mitotic activity (5 mitoses/10 HPF), composed of intersecting fascicles of spindle cells without significant atypia or necrosis (hematoxylin and eosin, ×200). (B) Myxoid leiomyosarcoma with abundant myxoid stroma and scattered atypical smooth muscle cells exhibiting pleomorphism and brisk mitotic activity (H&E, ×200). (C) Inflammatory leiomyosarcoma with diffuse infiltration of lymphocytes and eosinophils amidst pleomorphic spindle cells (H&E, ×200). (D) High-power view of inflammatory leiomyosarcoma highlighting marked cytological atypia, increased mitotic figures (15/10 HPF), and inflammatory background (H&E, ×400).

Histopathology showed abundant myxoid stroma, pleomorphic nuclei, >20 mitoses per 10 HPF, and necrosis (Figure 3B). IHC confirmed smooth muscle differentiation. A diagnosis of myxoid leiomyosarcoma was made. Despite surgical resection, the patient developed pulmonary metastases within 10 months and is currently undergoing chemotherapy.

Case 5: Inflammatory leiomyosarcoma

A 58-year-old woman presented with chronic pelvic discomfort, fatigue, and weight loss. CT scan revealed a 7 cm poorly circumscribed mass involving the uterine fundus and extending into the myometrium. Histological examination showed a spindle cell tumor with dense lymphocytic and eosinophilic infiltrates, high mitotic activity (Figures 3C-3D), and positive immunostaining for SMA and desmin. These findings supported the diagnosis of inflammatory leiomyosarcoma. The patient is currently receiving adjuvant therapy and remains under close oncological follow-up.

## Discussion

**Figure 4:**
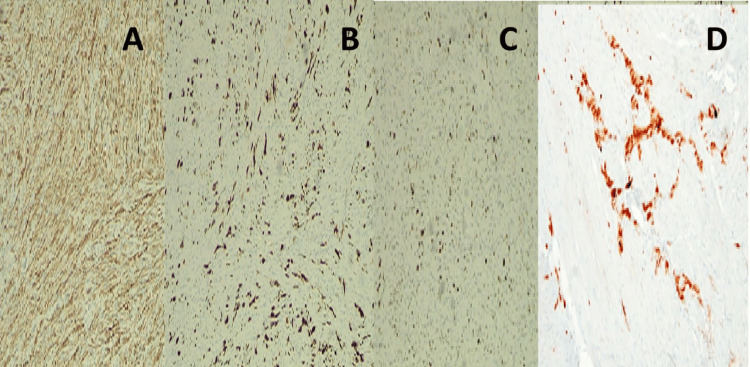
Immunohistochemistry findings. (A) Immunohistochemical stain showing diffuse cytoplasmic positivity for smooth muscle actin (SMA) in leiomyoma, confirming smooth muscle origin (IHC, ×200). (B) Desmin immunostaining highlighting smooth muscle fibers in a case of inflammatory leiomyosarcoma (IHC, ×200). (C) High Ki-67 proliferation index (>30%) in myxoid leiomyosarcoma, supporting malignant potential (IHC, ×400). (D) Focal nuclear positivity for calretinin in UTROSCT, aiding distinction from other uterine neoplasms (IHC, ×200). Images obtained with institutional permission from the Department of Pathology, NMC Royal Hospital, Sharjah. All patient identifiers removed. Use authorized for publication.

These cases highlight the broad histological and clinical range of uterine smooth muscle tumors. Benign variants, such as leiomyoma with increased mitoses, may not require aggressive treatment, whereas lesions, such as STUMP and leiomyosarcoma, necessitate close monitoring due to unpredictable behaviour [1,3]. UTROSCT, although rare, often exhibits indolent behavior but requires accurate identification due to its unique immunophenotype [4]. IHC is an important clue to subclassification and diagnosis of uterine smooth muscle tumors (Figure 4). Even though hematoxylin and eosin (H&E) morphology forms the basis of diagnosis, IHC brings clarity to lineage and rules out mimics [1,2]. Smooth muscle markers such as smooth muscle actin (SMA), desmin, and h-caldesmon are important to establish myogenic differentiation. Contrarily, ectopic expression of markers such as CD10, estrogen receptor (ER), and progesterone receptor (PR) is seen in variants. For tumors such as UTROSCT, positivity for sex cord markers (inhibin, calretinin, SF-1) and absence of muscle markers is in favor of the diagnosis. Ki-67 proliferation index and p53 staining assist in assessing aggressive potential, especially in STUMP and leiomyosarcomas [3,5]. Therefore, an optimal IHC panel, interpreted in conjunction with morphology, is crucial for accurate diagnosis and prognostication.

Histopathology remains the cornerstone of diagnosis, but immunohistochemical profiling is critical, especially in distinguishing UTROSCT and subtypes of leiomyosarcoma. Smooth muscle markers (SMA, desmin) confirm muscle origin, while sex cord markers (inhibin, calretinin) help classify UTROSCT [4,5].

An important aspect in the evaluation of uterine smooth muscle tumors is the recognition of diagnostic pitfalls. Certain benign variants, such as leiomyomas with increased mitotic activity, can mimic malignancy, whereas rare subtypes, such as STUMP or myxoid leiomyosarcoma, may initially appear deceptively indolent. Misinterpretation can lead to under- or overtreatment, underscoring the need for careful correlation of histomorphology, IHC, and clinical context [1,3]. For example, UTROSCT may be misclassified as other epithelial or stromal neoplasms without appropriate sex cord marker staining, while epithelioid leiomyosarcomas require recognition of nuclear atypia and mitotic activity to guide prognosis [4,5]. From a management perspective, accurate subclassification informs surgical planning, the need for adjuvant therapy, and follow-up strategy, with aggressive tumors requiring multidisciplinary coordination and closer surveillance [5,6]. Highlighting these diagnostic challenges alongside management considerations ensures that the findings serve as a practical reference for both pathologists and gynecologists.

Compared to existing literature, our cases reflect the diagnostic complexity of rare uterine smooth muscle tumors. For instance, Croce et al. described the aggressive clinical behavior of myxoid leiomyosarcoma, aligning with our Case 4, where metastasis occurred within 10 months after surgery [5]. Similarly, the immunohistochemical profile of UTROSCT in our Case 2 is consistent with the findings reported by Pradhan et al., who emphasize the utility of calretinin and inhibin in distinguishing these rare tumors [4].

The diagnosis of STUMP remains ambiguous, as shown in the Guntupalli et al. study, where recurrence was observed despite low mitotic activity [3]. STUMP can be classified as (1) uterine spindle cell STUMP: only one morphologic criterion of malignancy, (2) myxoid STUMP: mitoses (1/10 high power fields in the absence of cytologic atypia and tumour cell necrosis), and (3) epithelioid STUMP: mitoses (≥ 2/10 high power fields and < 4/10 high power fields in the absence of cytologic atypia and tumour cell necrosis). Our STUMP case mirrors this risk, underscoring the need for long-term follow-up [3]. These case correlations highlight the importance of individualized diagnostic algorithms based on histology, IHC, and clinical context. Table 1 presents the key differential diagnoses of uterine mesenchymal tumors.

**Table 1:** Key differential diagnoses of uterine mesenchymal tumors. IHC: immunohistochemistry; HLRCC: hereditary leiomyomatosis and renal cell cancer; SMT: submucosal tumors; STUMP: smooth muscle tumors of uncertain malignant potential; UTROSCT: uterine tumors resembling ovarian sex cord tumors

Entity	Key Morphological Features	Helpful IHC Markers	Notes
Leiomyoma/variants	Spindle cells, fascicular pattern, minimal atypia	SMA, desmin, h-caldesmon	Mitotic count important
STUMP	Borderline features, focal atypia	Variable: p53, Ki-67 (adjunctive)	Unpredictable behaviour
Leiomyosarcoma	Marked atypia, high mitoses, necrosis	SMA, desmin; high Ki-67	Aggressive clinical course
Endometrial stromal neoplasm	Infiltrative growth, arterioles	CD10, ER, PR	Common mimic of SMT
UTROSCT	Sex-cord-like architecture	Inhibin, calretinin, SF-1	Usually indolent
PEComa	Epithelioid cells, clear cytoplasm	HMB45, Melan-A, SMA	Rare, dual lineage
FH-deficient leiomyoma	Prominent nucleoli, perinucleolar halos	FH loss, 2SC-positive	Consider HLRCC
Epithelioid leiomyosarcoma	Epithelioid cells, necrosis	SMA ±, desmin variable	Different diagnostic criteria

An important and well-recognized diagnostic challenge in uterine mesenchymal pathology is the distinction between uterine smooth muscle tumors and endometrial stromal neoplasms. These entities frequently overlap morphologically on routine H&E sections, particularly in cases showing spindle cell morphology, infiltrative growth patterns, and variable mitotic activity. As a result, misclassification is a common pitfall in routine practice and highlights the need for careful correlation with IHC and clinical findings [7,8]. While smooth muscle tumors typically demonstrate expression of SMA, desmin, and h-caldesmon, endometrial stromal tumors more often show CD10 positivity along with ER and PR expression, aiding lineage assignment [1,2].

In addition to endometrial stromal neoplasms, a number of less common uterine mesenchymal tumors should also be considered in the differential diagnosis. These include perivascular epithelioid cell tumor (PEComa), fumarate hydratase (FH)-deficient leiomyoma, and an expanding group of molecularly defined uterine sarcomas. PEComas may closely mimic epithelioid smooth muscle tumors histologically but are characterized by dual expression of melanocytic markers (HMB45, melan-A) and smooth muscle markers, which is diagnostically helpful [9]. FH-deficient leiomyomas, increasingly recognized due to heightened awareness of HLRCC syndrome, show distinctive morphological features, such as prominent nucleoli and perinucleolar clearing, supported by loss of FH expression or positive 2SC staining [10].

Furthermore, epithelioid leiomyosarcoma represents a distinct subtype with diagnostic criteria that differ from conventional leiomyosarcoma. In these tumors, cytologic atypia and tumor cell necrosis may carry greater diagnostic weight than mitotic activity alone, underscoring the importance of recognising subtype-specific features to avoid under- or over-diagnosis [1]. Taken together, these considerations emphasize that uterine mesenchymal tumors represent a heterogeneous and evolving group, where accurate classification relies on a systematic approach integrating morphology, IHC, and selective molecular studies. Such an approach is essential to ensure correct diagnosis, guide appropriate clinical management, and inform prognostication.

Management strategies depend on histological diagnosis and biological behavior. While benign tumors require minimal follow-up, aggressive sarcomas call for multidisciplinary management, including surgery, chemotherapy, and regular imaging surveillance [5,6].

## Conclusions

Uterine smooth muscle tumors encompass a diverse pathological spectrum, and accurate classification using detailed histological and immunohistochemical analysis is essential for guiding appropriate management. This case series highlights rare variants, emphasizes relevant prognostic factors, and outlines follow-up considerations tailored to each tumor type. While our findings contribute to the limited literature and support a personalized approach to clinical care, the small case number and rarity of these tumors are recognized as limitations, underscoring the need for ongoing data collection and long-term studies.
